# Isoflavone Content and Nutritional-Related Properties of Debittered Seeds from Two Andean Lupin (*Lupinus mutabilis* Sweet) Ecotypes Propagated in Two Soils

**DOI:** 10.3390/foods12091841

**Published:** 2023-04-28

**Authors:** Francisco Urrego-Pava, Ericsson Coy-Barrera

**Affiliations:** Bioorganic Chemistry Laboratory, Universidad Militar Nueva Granada, Cajicá 250247, Colombia; francisco.urrego@unimilitar.edu.co

**Keywords:** Fabaceae, *Lupinus mutabilis*, isoflavones, antioxidant activity, functional foods

## Abstract

*Lupinus mutabilis* Sweet is a fabaceous plant native to the Andean highlands and produces seeds with valuable nutritional properties. Thus, as part of our research on native emerging food, the present study aimed at determining some nutritional and functional-related features of seeds from two *L. mutabilis* ecotypes after propagation in two different substrates commonly found in the Bogotá plateau. Propagated plants produced seeds that, after conventional debittering, exhibited attractive contents of soluble protein (24–39 g/100 g dry seed powder (dsp)), phenolic (787–1003 g/100 g dsp), isoflavone (1–104 g/100 g dsp), and iron (5.3–6.4 g/100 g dsp), as well as antioxidant capacity (39–78 µM/100 g dsp). Higher pH, humidity saturation, organic matter, and total nitrogen of silty loam soil promoted isoflavone accumulation and better antioxidant capacity at pH 4–7, and no soil effect was observed for total phenolic and iron contents. The profiles based on isoflavone aglycones were also recorded by liquid chromatography-mass spectrometry, detecting eleven main compounds with mutabilein as the most abundant isoflavone (38.3–104.3 g/100 g dsp). Finally, a formulation was developed to fabricate an emulsion-type drink based on the debittered, pulverized *L. mutabilis* seeds, resulting in different emulsifying capacities (19–100%) depending on the biopolymer stabilizer, being xanthan gum the best additive. The findings revealed an attractive Andean lupin profile to be used as a raw food material.

## 1. Introduction

Functional foods have been highly relevant for centuries since they contain biologically active compounds, have beneficial health properties, and can be used to treat or prevent diseases [1]. This kind of food has a long history, but it has had growing global demand since the 1990s [2]. Examples of relevant functional foods are related to legume seeds, such as lupins, which have many uses due to their significant nutritional value for humans and animals [3,4]. In this context, seeds of *Lupinus mutabilis* Sweet, a fabaceous plant native to the Andean highlands, have exhibited attractive properties [5]. These seeds have been used daily for nutritional and health-beneficial purposes by indigenous communities for more than 1800 years in territories comprising Peru, Ecuador, and Bolivia since they have a good content of protein, fatty acids, and bioactive compounds (e.g., isoflavones, carotenoids, phytosterols) [5] and antioxidant activity [6]. Although *L. mutabilis* seeds were employed for centuries by Andean native people [5], it is not relevantly exploited in Colombia [7].

Isoflavones are naturally occurring compounds produced by the specialized metabolism of legumes, and although they exist in other species, they have only been reported in a small group of bacteria and fungi [8,9]. In plants, these metabolites are primarily produced in the roots and seeds and satisfy essential functions, e.g., they are involved in nodulation mechanisms [10] and defense against pests and pathogens [11]. In addition, isoflavones may benefit humans due to their estrogenic activity [12], but also as immunostimulatory agents in cancer prevention, cardiovascular disease, osteoporosis, obesity, irritable bowel syndrome symptoms, and as antioxidants [8,13,14,15,16]. These facts have suggested that isoflavones are relevant as health promoters in human nutrition [17]. Therefore, those isoflavone-producing foods are highly desirable [18,19]. Other nutritional parameters for attractive gluten-free foods are related to the iron and protein content due to their importance for human health [20,21], and *L. mutabilis* seeds can cover such supplies for good nutrition [5]. In this context, chemical parameters and seed quality of some *Lupinus* plants can vary depending on ecotypes, environmental conditions, and soil requirements and features due to their genetic variability and metabolic-based phenotypic plasticity [22,23,24,25], which deserve further exploration to outline other traits for agronomic performance and breeding of *L. mutabilis* as emerging food and crop [26,27].

Therefore, as part of our research on the properties of native Andean foods, the present study was focused on the chemical characterization of conventionally debittered seeds from two Andean lupin ecotypes after propagation on two typical soils of the Andean highlands in Bogotá plateau, Colombia. This characterization was focused on nutritional (soluble protein and iron) and functional (antioxidant capacity, phenolic content, and isoflavone profiles) properties. Thus, soluble protein, iron, and phenolic contents and antioxidant capacity were measured by colorimetric-based methods, whereas isoflavone aglycones were analyzed by liquid chromatography coupled with mass spectrometry after acid hydrolysis and hydroalcoholic extraction. The findings illustrated Andean lupin seeds’ adaptive potential, although certain variability of some parameters was evidenced. Our findings led to defining these soil-dependent variations as value parameters and traits for these seeds derived from *L. mutabilis* ecotypes.

## 2. Materials and Methods

The workflow of this study was divided into three phases. The first phase was oriented to propagate two *L. mutabilis* ecotypes (from Cajicá and Pasto) using two different soils (silty loam and sandy clay loam). The second phase focused on the chemical characterization of the harvested seeds from two ecotypes and two soils (2 × 2), starting with the initial soluble protein content measurements, then subjecting the seed to a quinolizidine alkaloid elimination process (i.e., debittering) and, finally, quantifying the residual alkaloid content. Subsequently, the soluble protein, phenolics, isoflavone, and iron contents and the antioxidant capacity of the free-alkaloid seed powders were determined. The third phase was finally developed to explore a formulation of an *L. mutabilis* seed-based drink as a value-added product, constituting an alternative for small to medium producers.

### 2.1. Phase I: L. mutabilis Propagation

#### 2.1.1. Plant Material

The present study employed *L. mutabilis* Sweet seeds obtained from existing plants in two Andean locations. Thus, the first material was obtained from the in-house *L. mutabilis* collection grown in the greenhouses at the Universidad Militar Nueva Granada (UMNG) Campus, Cajicá, Colombia, namely as LC seeds. The “*Red de Guardianes de Semillas de Vida*” provided the second seed material from *L. mutabilis* plants cultivated at Pasto, Colombia, and denoted as LP seeds. Cajicá and Pasto have subtropical highland climates (i.e., Cfb, according to the Köppen-Geiger system), including average temperature, annual precipitation, and altitude of 14.1 vs. 11.2 °C, 1493 vs. 2135 mm, and 2560 vs. 2535 m above sea level (masl), respectively. However, although LC and LP seeds shared a white coloration, they differed by some features, average mass, and mean size. They can be oval, flattened, spherical, like the lentil seed (*Lens culinaris*), or spherical and round. LC seeds had an average diameter of 9.93 ± 0.45 mm (mean ± standard deviation, SD, *n* = 30) and an average dry weight of 0.33 ± 0.03 g. LP seeds have an average diameter of 9.11 ± 0.20 mm and an average dry weight of 0.27 ± 0.02 g. Owing to the exhibited statistically significant differences (*p* < 0.05) in these parameters, they were considered *L. mutabilis* ecotypes.

#### 2.1.2. Germination and Propagation

One-hundred LC and one hundred LP seeds were sown in a seedbed with substrate prepared from 50% peat and 50% silty loam soil and arranged inside a greenhouse for germination. Each seedbed was placed on a tray that retained water two centimeters above the bottom to guarantee the necessary humidity for the seeds, and the water amount was controlled every two days. After 30 days from sowing, the seedlings were placed into a 2 kg polyethylene bag. The plants were maintained inside the greenhouse at UMNG (coordinates = 4°56′ N, 74°00′ W, altitude = 2560 masl, temperature = 20 ± 5 °C; relative humidity (RH) = 68 ± 14%, total light transmission = 85 ± 5%, total light diffusion = 57 ± 5%, and UV transmission between 290–340 nm = 6%). The humidity was kept with a tray under the plants, and the water was controlled every two days. After 120 days of seed sowing (>94% germination for both ecotypes), the 30 highest LC and LP plants (ca. 12 cm height) were selected and transplanted into two different soils, i.e., silty loam (sl) and sandy clay loam (scl) soils, whose characteristics are presented in Table A1 in Appendix A.

The plants were sown following a 2 × 2 arrangement (i.e., two ecotypes grown in two soils). Therefore, fifteen LC plants in sl soil produced LC-sl seeds; fifteen LP plants in sl soil produced LP-sl seeds; fifteen LC plants in scl soil produced LC-scl seeds, and, finally, fifteen LP plants in scl soil produced LP-scl seeds. Hence, sixty plants were planted in sixty 0.15 mm thick polyethylene bags containing 20 L of the test soils and organized in four rows comprising test ecotypes and soils. Each plant row was staked out with a 3 mm diameter plastic rope to prevent the propagated *L. mutabilis* plants from being deflected or knocked down by the wind. Irrigation was carried out thrice weekly, and no pesticides or agrochemicals were used. After 145 ± 9 days after transplanting, the 30 LC plants and 30 LP plants were harvested to collect ca. 500 g of seeds. The seed collection was done when a free-seed-related sound was heard inside on moving pod.

### 2.2. Phase II: Chemical Characterization of L. mutabilis Seeds Harvested from Propagated Plants

#### 2.2.1. Raw Seed Protein Determination

Protein determination was performed from the raw and previously ground LC-sl, LP-sl, LC-scl, and LP-scl seeds using the reported method with slight modifications [28]. Thus, seed powder (1 g) was deposited in a 100 mL beaker, and 1% NaCl (10 mL) was added. The dispersion was subjected to magnetic stirring for 10 min, then centrifuged for 5 min at 1000 g. The supernatant was deposited in a 25 mL volumetric flask. The solid residue was again treated with 1% NaCl solution (10 mL), stirred, and centrifuged. The resulting supernatants were mixed into the flask, and the volume was finally completed to the mark. The soluble protein determination was performed using Bradford reagent in a VariosKan LUX microplate reader (Thermo Fischer Scientific, Waltham, MA, USA) on a 96-well plate (220 µL per well) as follows: bovine serum albumin (BSA) (5 mg) was mixed with 1% NaCl (100 mL) and used to build a calibration curve (0, 10, 25, 50, 60, 75, 90, and 100%). The wells were then filled with the test extract (10 µL), 1% NaCl (55 µL), and Bradford reagent (120 µL). The absorbance was measured at 595 nm, and soluble protein content was expressed as % protein (dry basis). Determinations were performed by using three biological and three technical replicates. The analytical method performance was monitored for each analysis batch, involving five quality control (QC) samples spiked with 10% BSA to assess the method response variations (coefficient of variation (CV) < 5%).

#### 2.2.2. Raw Seed Hydroalcoholic Extraction under Acidic Hydrolysis

A protocol oriented to the extraction of (iso)flavone aglycones was followed [29] to characterize chemically the seeds (i.e., LC-sl, LP-sl, LC-scl, and LP-scl) obtained from propagated *L. mutabilis* plants. Briefly, harvested seeds (10 g) from two *L. mutabilis* cultivated in the test two soils were dried in an oven at 40 °C for 96 h, cooled in a desiccator for 1 h, and ground using a Pulverisette mill (Fritsch GmbH, Idar-Oberstein, Germany) equipped with a 0.12 mm particle size blade. The resulting seed powder (75 mg) was placed in a centrifuge tube, and 80% ethanol (4.5 mL) was added. The solution was acidified with 1 M HCl, incubated at 80 °C in a hot water bath for 60 min, allowed to cool, strongly vortexed for 10 min, and centrifuged at 1500 g for 10 min. The solid residue was again extracted with 80% ethanol, stirred, and centrifuged. The resulting supernatants were combined, neutralized, and placed into a 10 mL volumetric flask. The volume was finally completed to the mark with 80% ethanol. The resulting solutions were transferred to blue-lid bottles and stored at −20 °C until chemical analyses.

#### 2.2.3. Alkaloid Removal Process (Debittering)

*L. mutabilis* seeds (100 g) were gathered and soaked using a 1:4 seed/water ratio (i.e., 100 g seeds per 400 mL water). After an 18 h soaking, the alkaloid-containing water was discarded and replaced with fresh water (1:3 seed/water ratio), and, finally, the seed residue was boiled for 1 h [30]. Two extraction conditions were examined at this stage:

I. neutral extractant, i.e., the boiling water was replaced by fresh recirculating water (1:3 seed/water ratio), using a Daihan Maxircu-CH 12 bath circulator (DKSH Holding Ltd., Zurich, Switzerland), and the seed residue was maintained for several h at 16 °C until alkaloid depletion. The water was changed three times a day (i.e., every 8 h), using the 1:3 seed/water ratio. Plastic mesh bags (0.5 mm mesh) were used to store the seeds during extraction. The resulting alkaloid-free seeds were labeled with the subscript acronym “af” (i.e., LC_af_-sl, LP_af_-sl, LC_af_-scl, LP_af_-scl, LP_af_-sl) to code the alkaloid removal.
II. acidic extractant, i.e., the boiling water was replaced by 0.5 M citric acid solution. Extraction conditions were identical to those described for water (neutral extraction). This acidic process was only performed with LC-sl seeds for comparative purposes, and the resulting extract was denoted as LC_af_-sl + A.

The recirculation (using water or an acidic solution) was maintained until the resulting aqueous solution did not produce red–orange color on the SiO_2_-containing thin-layer chromatography (TLC) plate due to the alkaloid presence by using the Dragendorff reagent. After this point (>130 h), the seed residues were dried (oven, 40 °C, 96 h) and cooled in a desiccator for 1 h. The dry seed weight was then recorded, and the resulting material was milled using the Pulverisette mill for subsequent hydroalcoholic extraction.

#### 2.2.4. Alkaloid Extraction for Quantitative Purposes

A previously described procedure was followed for the alkaloid extraction [31]. Thus, the dried ground seed material (75 mg) and 0.5 M HCl (10 mL) were added to a flask. The mixture was vigorously stirred for 10 min by vortex and centrifuged for 10 min at 4200 g. The pellet was again resuspended in 0.5 M HCl (10 mL) and centrifuged. The supernatants were combined, alkalinized with 4 M NH_4_OH (pH 12–14) for 15 min, and extracted with dichloromethane. The organic phase was concentrated using a rotary evaporator at 40 °C, transferred to a vial, dried, and stored at –20 °C until gas chromatography coupled with mass spectrometry (GC-MS) and flame ionization detector (GC-FID) analyses. On the other hand, recirculated water after debittering, when the orange-red color was not appreciable under the Dragendorff test in the TLC plate, was also analyzed to determine the limit of detection (LoD) of this method. Thus, aliquots (25 mL) of the different rounds of recirculated water were concentrated under reduced pressure at 40 °C. The resulting residue was dried and stored at −20 °C until GC-MS analysis.

#### 2.2.5. GC-MS and GC-FID Analyses

The GC-MS analysis was performed to detect and identify alkaloids using a Thermo Trace 1300 gas chromatograph coupled with an ISQ LT mass spectrometer with a single quadrupole analyzer, with an RXi 5Sil MS (5% diphenyl, 95% dimethylpolysiloxane) column (60 m × 0.25 mm, 0.25 μm film thickness) (Restek Corp., Bellefonte, PA, USA). The temperature program was employed as follows: the starting temperature was 40 °C for 1 min, then a 6 °C/min program until 290 °C and retained for 6 min. The transfer line temperature was 250 °C, and the carrier gas was grade-5 helium (flow = 1 mL/min). The ionization mode was the electronic impact (EI) at 70 eV. The alkaloid quantification was performed by GC-FID under identical GC conditions using (+)-lupanine (Sigma-Aldrich, St. Louis, MO, USA) as an external standard. The alkaloid contents were expressed as mg lupanine equivalents per 100 g of dry seed powder (mg eq lupanine/100 g dry seed powder (dsp)). Peak areas of detected alkaloids were corrected with the relative response factors. Determinations were performed by using three biological and three technical replicates. The method precision was evaluated through intra and inter-day lupanine analyses, whose relative standard deviations (RSD%) were 4.2 and 3.4%, respectively. The LoD, limit of quantification (LoQ), and recoveries for the GC-FID lupanine analysis were 1 µg/mL, 2 µg/mL, and 95.5–104.1%, respectively [32]. QC pooled samples were analyzed to assess the detector response variation (CV < 5%).

#### 2.2.6. Protein Determination of Alkaloid-Free Seeds

Protein determination was performed with previously milled, alkaloid-free *L. mutabilis* seeds (i.e., LC_af_-sl, LP_af_-sl, LC_af_-scl, LP_af_-scl, LP_af_-sl, and LC_af_-sl + A). This determination used the identical procedure described in Section 2.2.1.

#### 2.2.7. Hydroalcoholic Extraction from Alkaloid-Free *L. mutabilis* Seeds

The hydroalcoholic extracts from the milled, alkaloid-free *L. mutabilis* seeds (i.e., LC_af_-sl, LP_af_-sl, LC_af_-scl, LP_af_-scl, LP_af_-sl, and LC_af_-sl + A) were prepared following the extraction protocol for hydrolyzed isoflavones described in Section 2.2.2. Since isoflavones are phenolics with antioxidant activity, the resulting extracts obtained from this protocol were also used to determine the total phenolic content and the radical scavenging capacity.

#### 2.2.8. Total Phenolic Content

The total phenolic content was determined through the Folin–Ciocalteau reagent [33]. Briefly, a solution of the test extracts (20 μL), 10% Folin–Ciocalteau reagent (40 μL), and 7.35% sodium carbonate (140 μL) were added to a well of a 96-well plate. The blank comprised deionized water (20 μL), 10% Folin–Ciocalteau reagent (40 μL), and 7.35% sodium carbonate (140 μL). The mixture reacted in the dark for 1 h, and then absorbance was measured at 765 nm using a Thermo Scientific Varioscan LUX microplate reader (Thermo Fischer Scientific, Waltham, MA, USA). The absorbance values were converted to total phenolic content using a calibration curve previously constructed with a gallic acid standard (Sigma-Aldrich, St. Louis, MO, USA). Thus, the total phenolic contents were expressed as mg gallic acid equivalents per gram of seed powder (mg eq gallic acid/100 g dsp). Determinations were performed by using three biological and three technical replicates. The analytical method performance was monitored for each analysis batch, involving five QC samples spiked with 10% gallic acid to assess the method response variations (CV < 5%).

#### 2.2.9. LC-MS Analysis of Seed-Derived Extracts

The chemical profiles of raw and alkaloid-free seed-derived extracts were recorded by liquid chromatography coupled with mass spectrometry (LC-MS). For this purpose, a Prominence Ultra-Fast Liquid Chromatographic (UFLC) system (Shimadzu, Columbia, MD, USA), equipped with a photodiode array (PDA) detector and a Shimadzu LCMS 2020 mass spectrometer with quadrupole analyzer and electrospray ionization (ESI) operated in positive and negative ion modes, was then used. The separation was performed on a Synergi C18 column (150 × 4.6 mm, 4 µm) (Phenomenex, Torrance, CA, USA), injecting 10 µL of the sample, and using a mixture of solvents A (1% formic acid in water) and B (1% formic acid in acetonitrile). The gradient elution method started with 0–1 min 5% B, 1–12 min 5% to 40% B, 12–15 min 40% B, 15–19 min 40% to 100% B, 19–22 min 95%, and 22–25 min 95% to 5% B). This detection was combined by high-resolution MS (HRMS) recorded on an Agilent Technologies 1260 Liquid Chromatograph coupled with a quadrupole-time-of-flight (Q-ToF) mass analyzer with dual Agilent jet stream electrospray ionization (AJS-ESI) (Agilent, Santa Clara, CA, USA). Chromatographic analysis was performed under identical conditions to those afore mentioned. The AJS-ESI ionization was operated in negative ion mode and involved a capillary voltage (3500 V), drying gas (8 L/min), gas temperature (325 °C), nebulizer pressure (50 psi), sheath gas temperature (350 °C), and sheath gas glow (11 L/min). The Q-ToF comprised fragmentor voltage (175 V), skimmer voltage (65 V), and octapole radiofrequency peak to peak voltage (OCT RF Vpp) (750 V). The UV-Vis, MS, and HRMS-derived data were used for compound annotation through the combined diagnostic analysis (i.e., λ_max_, accurate mass, quasimolecular ion, and MS fragments), supported by phylogeny, chromatographic performance, and comparison with literature and various databases, e.g., KNApSAcK [34], dictionary of natural products [35], and PubChem [36]. The isoflavone quantification was performed using genistein (Sigma-Aldrich, St. Louis, MO, USA) as an external standard and PDA detector monitoring at 325 nm. The isoflavone contents were expressed as mg genistein equivalents per 100 g of dry seed powder (mg eq genistein/g dsp). Peak areas of detected (iso)flavonoids were corrected with the relative response factors. Determinations were performed by using three biological and three technical replicates. The method precision was assessed by the intra and inter-day analyses of genistein, whose relative standard deviations (RSD%) were 3.1 and 3.9%, respectively. The LoD, LoQ, and recoveries for the LC-DAD genistein analysis were 440 ng/mL, 920 ng/mL, and 94.1–104.6%, respectively. The total isoflavone content was afforded through the sum of the contents of individual compounds. Pooled samples as QC were injected to assess the detector response variation (CV < 5%).

#### 2.2.10. Antioxidant Capacity

The diphenylpicrylhydrazil radical (DPPH^•^) scavenging method was used to determine the antioxidant capacity of test seed materials [33]. Briefly, test extract solution (20 μL), 10 μM DPPH solution (150 μL), and absolute ethanol (50 µL) were added to a well of a 96-well plate. The blank involved absolute ethanol (70 µL) and 10 mM DPPH solution (150 μL). The mixture reacted for 1 h in the dark, and the absorbance was measured at 515 nm using a Varioscan LUX microplate reader (Thermo Fischer Scientific, Waltham, MA, USA). In addition, the antioxidant capacity was also determined at different low pH values (from 1 to 7) using identical conditions. The pH was modified for the test extracts using buffers to get the desired pH value. The absorbance values were converted to Trolox equivalent antioxidant activity (TEAC) using a calibration curve previously constructed with a Trolox standard (Thermo Fischer Scientific, Waltham, MA, USA). Thus, the TEAC values were expressed as µM Trolox per 100 g dry seed powder (µM/100 g dsp). Determinations were performed by using three biological and three technical replicates. The analytical method performance was monitored for each analysis batch, involving five QC samples spiked with 10% Trolox to assess the method response variations (CV < 5%).

#### 2.2.11. Iron Content

The iron contained in the seed was determined by spectrophotometry using the ortho-phenanthroline (OPT) method of the Association of Official Analytical Chemists (AOAC) [37]. A calibration curve was firstly constructed. Thus, a 1000-ppm ferrous ammonium sulfate hexahydrate (FASH) stock solution was used as a stock solution. From this solution, 5 mL were retrieved and taken to the mark of a 500 mL volumetric flask to obtain a 10-ppm standard solution (SS). Eight working solutions (WS) were prepared in 100 mL volumetric flasks and marked as WS 2, 5, 10, 15, 20, 30, and 40. Thus, 2 mL of the 10-ppm FASH SS were added to the WS 2, 5 mL of the FASH SS were added to WS 5, 10 mL of the FASH SS were added to WS 10, and thus, the respective amounts of reagent were added to each WS flasks. No FASH was added to the blank. Subsequently, concentrated HCl (2 mL) and 10% hydroxylamine hydrochloride solution (1 mL) were added to each flask. It was incubated for 5 min. A pH = 3 was achieved by adding sodium acetate/glacial acetic acid buffer solution (22 mL, pH 4.7), and, finally, 1% OPT solution (6 mL) was added. Each flask was calibrated with distilled water, carefully agitated, and incubated for 30 min. The absorbance was determined at 510 nm.

For the iron quantification in *L. mutabilis* seeds, alkaloid-free seeds (1.5 g) were initially placed into a crucible, which was heated at 550 °C for 5 h in a muffle. After this time, the ashes were left to cool in a desiccator. Subsequently, concentrated HCl (5 mL) was added to each cold crucible and heated at 80 °C in an extractor cabinet until acid evaporation. Concentrated HCl (2 mL) was again added, and the acid was again allowed to evaporate. Then, the crucible was allowed to cool to room temperature, distilled water (5 mL) was added, and the solution was completed to the mark of a 50 mL volumetric flask with distilled water. From each resulting solution, 10 mL were taken with a volumetric pipette and transferred to a 100 mL volumetric flask. 10% hydroxylamine hydrochloride (1 mL) was added and incubated for 5 min. The pH = 3 was adjusted by adding sodium acetate/glacial acetic acid buffer solution (10 mL), and 1% OPT (6 mL) solution was added. The final volume was completed to the flask mark with distilled water, carefully agitated, and incubated for 30 min. The absorbance was measured at 510 nm. The results were expressed as mg Fe^2+^ per 100 g of dry seed powder (mg Fe^2+^/100 g dsp). Determinations were performed by using three biological and three technical replicates. The analytical method performance was monitored for each analysis batch, involving five QC samples spiked with 20% FASH to assess the method response variations (CV < 5%).

### 2.3. Phase III: Determination of the Emulsifying Capacity of a Lupin-Seed-Based Drink Formulation

Three emulsions were prepared in triplicate [38] based on soy beverage formulations [39]. Briefly, nine 150 mL beakers were filled with distilled water (93 mL) and heated at 60 °C on a heating plate with magnetic stirring at 200 rpm. Coconut fat (1.5 g), alkaloid-free lupin seed flour (8 g, equivalent to 2.8 g protein), sucrose (2 g), palm-distilled monoglycerides (0.2 g), NaCl (0.1 g), and sodium azide (0.03 g) as a preservative were added. Gellam gum (0.05 g) was added to three beakers, carrageenin (0.3 g) was added to the other three beakers, and xanthan gum (0.2 g) was finally added to the remaining three beakers. After mixing all the ingredients, the stirring was prolonged for 3 min, and the resulting dispersions were then cooled down to room temperature and stored in covered bottles at 4 °C for 18 h. After this time, the coconut fat floating on the dispersion was freeze-dried and gravimetrically measured to determine the emulsifying capacity of each formulation. Each sample was stored at 4 °C and checked 10, 20, 30, and 60 days after preparation to check the emulsion stability.

### 2.4. Statistical Analyses

A Shapiro–Wilks normality test was used to check the normal data distribution (*p* > 0.05). Once the normality was checked, an analysis of variance (ANOVA) was performed, followed by a post hoc Tukey test to define significant differences between means (*p* < 0.05). The inferential statistics were performed in InfoStat software [40]. In addition, the partial least square discriminant analysis (PLS-DA) was performed on LC-derived quantitative data using the statistical module within Metaboanalyst 5.0 web-based tool [41].

## 3. Results

### 3.1. Phase I: Propagation of Two L. mutabilis Ecotypes Using Two Soil Types

**Figure 1:**
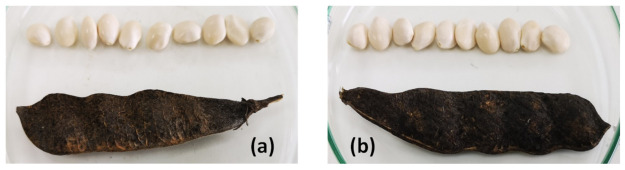
Seeds and pods of the test *Lupinus mutabilis* Pasto (LP) and Cajicá (LC) ecotypes propagated in silty loam (sl) soil. (**a**) *L. mutabilis* from Pasto (LP). (**b**) *L. mutabilis* from Cajicá (LC).

The propagation of *L. mutabilis* ecotypes (i.e., LC and LP) growing in two different soil types (i.e., sl and scl) comprised sixty plants (fifteen per ecotype and soil). All plants started flowering between weeks 12 to 14 after transplanting, and after week 20, the first seeds were harvested from mature plants of both ecotypes. Seed production was maintained for the next 12 months of crop monitoring. The collected seeds from the experimental crop exhibited identical characteristics to those used in the propagation stage. In addition, flowers produced by both ecotypes had the typical *L. mutabilis* color (white-patched purple-blue coloration). In general, no significant differences were observed in the plant growth performance according to the soil type. Thus, the LC plants reached an average height of 146 ± 6 cm and 143 ± 5 cm in sl and scl soils, respectively, while the LP plants had an average height of 117 ± 8 cm and 115 ± 9 cm in sl and scl soils, respectively. In addition, compared to the LC ecotype, LP exhibited a less robust structure in both soils and lower seed production. The LC ecotype also produced seeds in uniform dark brown pods, while LP ecotype pods exhibited a dark yellowish color with brown spots in both soils, which indicated phenotypic differences depending on the test ecotype (Figure 1).

Although the harvested seeds from both ecotypes exhibited a similar appearance (color and shape, Figure 1), the soil characteristics appeared to influence the pod and seed features since, apart from the ecotype-dependent differences, the pod length, seed dry weight, and seed diameter varied according to the soil type. In this regard, sl soil seemed to promote more seed dry biomass and pod and seed size than the scl soil (Table 1) since, although no significant differences were observed for the pod size and seed diameter between soil types, the mean values tended to be lower in scl soil than sl soil.

### 3.2. Chemical Characterization of Raw Seeds from Propagated L. mutabilis Plants

#### 3.2.1. Raw Seed Protein Determination

**Figure 2:**
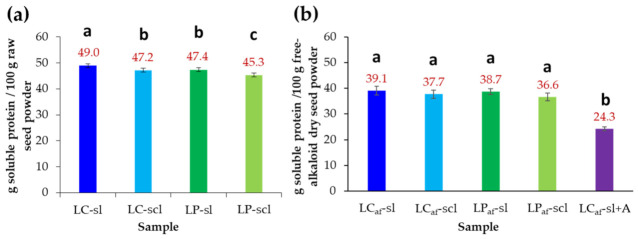
(**a**) Soluble protein content (determined by Bradford method) of raw seed samples of *L. mutabilis.* (**b**) Protein content of alkaloid-free seed samples of *L. mutabilis*. LC = *L. mutabilis* ecotype from Cajicá; LP = *L. mutabilis* ecotype from Pasto; LC-sl (LC ecotype grown on silty loam soil); LC-scl (LC ecotype grown on sandy clay loam soil); LP-sl (LP ecotype grown on silty loam soil); LP-scl (LP ecotype grown on sandy clay loam soil). Bars represent the mean values ± standard deviation (*n* = 6). Different lowercase letters over bars indicate statistically significant differences according to the post hoc Tukey test (*p* < 0.05).

The Bradford-determined protein content from the raw seeds of *L. mutabilis* produced by plants propagated in the two different soils was also determined and expressed as % *w*/*w* dry basis, i.e., grams of soluble protein per 100 g dry seed powder (g/100 g dsp). The measured soluble protein contents ranged from 45.3 to 49.0% (Figure 2a). Thus, the highest protein accumulation was observed for the LC-sl seeds (49.0% protein), while the lowest content was found for the LP-scl seed (45.3% protein). LC seeds accumulated more protein than LP seeds, but the seeds from both *L. mutabilis* ecotypes appeared to accumulate more protein content in sl than scl soil. In this sense, LC-sl seeds had 1.7% more protein than LC-scl seeds; and LP-sl seeds had 2% more protein than LP-scl seeds (Figure 2a).

#### 3.2.2. LC-MS Analysis of *L. mutabilis* Raw Seed-Derived Extracts

**Figure 3:**
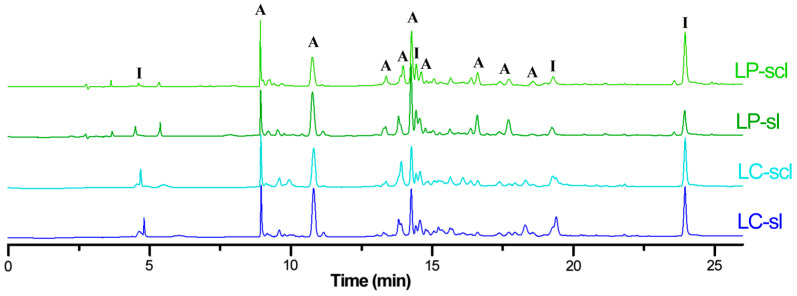
LC-MS-derived chromatograms of *Lupinus mutabilis* raw seed extracts obtained after hydroalcoholic extraction under acidic hydrolysis [29]. LC = *L. mutabilis* ecotype from Cajicá; LP = *L. mutabilis* ecotype from Pasto; LC-sl (LC ecotype grown on silty loam soil); LC-scl (LC ecotype grown on sandy clay loam soil); LP-sl (LP ecotype grown on silty loam soil); LP-scl (LP ecotype grown on sandy clay loam soil). Uppercase letters over signals indicate the compound type according to a preliminary MS-based annotation; I = (iso)flavonoid-related signal; A = alkaloid-related signal.

The hydroalcoholic extraction under acidic hydrolysis [29] was followed to remove the (iso)flavonoid aglycones from those seeds obtained from propagated *L. mutabilis* plants (i.e., LC-sl, LP-sl, LC-scl, and LP-scl). Subsequently, the resulting extracts were chemically characterized by LC-MS. Hence, Figure 3 shows the results obtained after LC-MS-based analysis of the raw seeds.

In these profiles, several signals were observed with slight differences because of the ecotype and soil type. However, chromatograms revealed several *m*/*z* signals related predominantly to those reported for alkaloids and their derivatives (annotated and labeled with A in Figure 3), so the extracted alkaloids appeared to mask the isoflavone-related profile, limiting their timely detection, identification, and quantification. This outcome led to eliminating alkaloids and focusing on isoflavone aglycones in the test seeds.

### 3.3. Alkaloid Removal Process and Protein Measurements in Alkaloid-Free L. mutabilis Seeds

The alkaloids were removed from the test *L. mutabilis* seeds after traditional debittering described in Section 2.2.3. Regarding the time and rounds of recirculation, the alkaloid removal protocol was optimized until GC-MS chromatograms did not show the residual presence of these compounds (alkaloid concentration in extracts <1 µg/mL = lupanine LoD). These contents lower than LoD were below 0.0025 mg eq lupanine/100 g dsp. In addition, the LoD of the Dragendorff test was found to be 48 µg/mL (0.06 mg eq lupanine/100 g dsp) in the resulting aqueous solutions after recirculating alkaloid removal when no red–orange spots were observed but analyzed by GC-MS.

The seed soluble protein content (PC) was again measured after alkaloid removal by water or citric acid. This removal revealed a ca. 10% protein reduction (Figure 2b). No significant differences were found between the seeds of the Cajicá and Pasto ecotypes grown on silt and sandy clay loam soils after debittering, although significant differences were found between the seeds debittered with water (>36.6%) and citric acid (24.3%). The PC comparison and other measurements before and after the debittering process are presented in Table 2. In this regard, the protein loss (PL) varied ca. 8.6% for LP seeds in both soil types, while 9.8 and 9.4% PL were exhibited for LC seeds in sl and scl soils. The highest PL was obtained after citric acid-based debittering, reaching 24.6% PL. However, although the PL was higher in LC than LP seeds, the dry matter loss (DML) exhibited an opposite trend since LC seeds lost ca. 27% DML, whereas LP seeds showed a ca. 32% DML for both soil types and neutral extraction. In contrast, citric acid-based extraction promoted the highest DML (38.1%).

The debittering finalized if alkaloid presence was below the LoD of the qualitative Dragendorff’s test; therefore, the whole debittering time (in h), and total water volume to remove alkaloids from 100 g of seeds differed between samples, i.e., 139–195 h and 4.9 to 7.0 L, respectively (Table 2). The neutral extraction was extended by 195 h and 7.0 L of water for LC-sl seeds since their total alkaloid content (TAC) was the highest (236.5 mg eq lupanine/100 g dsp), while LC-scl, LP-sl, and LP-scl seeds involved 187 h and 6.7 L (175.4 < TAC < 190.5 mg eq lupanine/100 g dsp). Citric acid-based extraction required less extraction time (139 h and 4.9 L) despite starting with the highest TAC but involved higher protein and matter loss.

### 3.4. LC-MS-Based Isoflavone Analysis

**Figure 4:**
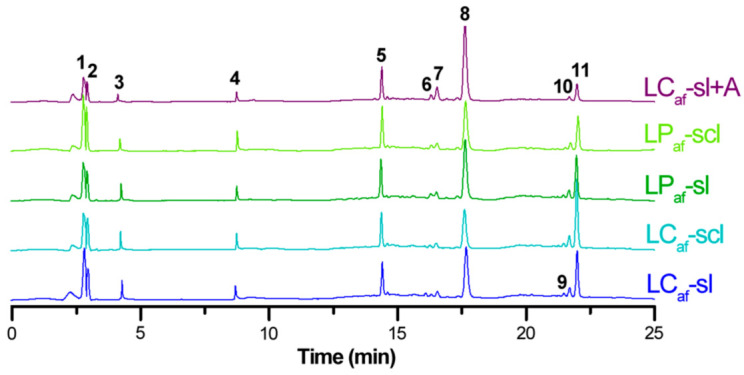
LC-MS chromatograms of alkaloid-free *Lupinus mutabilis* seed extracts obtained from the acid hydrolysis protocol [29]. LC = *L. mutabilis* ecotype from Cajicá; LP = *L. mutabilis* ecotype from Pasto; Subscript af abbreviation = alkaloid-free; LC_af_-sl (LC ecotype grown on silty loam soil); LC_af_-scl (LC ecotype grown on sandy clay loam soil); LP_af_-sl (LP ecotype grown on silty loam soil); LP_af_-scl (LP ecotype grown on sandy clay loam soil); LC_af_-sl + A (LC ecotype grown on silty loam soil and extracted with citric acid).

The LC-MS-based profiles of all resulting hydroalcoholic, acid-hydrolyzed extracts from those *L. mutabilis* seeds propagated in two soils revealed distinguishable chromatographic signals. These profiles shared the same peak number, indicating no additional compounds are differentially occurred by ecotype or induced by soil type (Figure 4). However, profiles had noticeable differences in the relative abundances to be further explored.

Eleven (iso)flavonoid aglycones (**1**–**11**) were detected and annotated for all seed extracts, whose annotation-leading diagnostic data are presented in Table 3. No alkaloids were detected in these analyses, exposing cleaner chemical profiles.

Ten compounds were related to isoflavones (**1**–**3** and **5**–**11**), and one compound comprised the flavone apigenin (**4**). The most representative compound appeared to be mutabilein (**8**), a known isoflavone previously isolated from *L. mutabilis* [43]. The other isoflavones were related to hydroxylated, methoxylated, and prenylated variants of mutabilein and genistein. The annotated (iso)flavone aglycones agreed with previously reported analysis on lupin plants [44,45].

The chromatographic separation exhibited a reasonable profile to be employed for quantitative purposes. Thus, the detected (iso)flavonoids **1**–**11** were quantitatively examined to assess the variations between collected *L. mutabilis* seeds. The quantitative levels of **1**–**11**, expressed as mg equivalent to genistein per 100 g of alkaloid-free dry seed powder (mg/100 g dsp), are shown in Table 4. The isoflavone content distribution is visualized by a heatmap-based scale (blue = high values; red = low values). The highest contents of the water-debittered sample set were evidenced for isoflavones **1**, **5**, **8**, and **11** (11.0–104.3 mg/100 g range), with **8** being the most abundant isoflavone (>38 mg/100 g), and the lowest contents resulted for **3**, **6**, and **9** (<6.2 mg/100 g). However, this global distribution changed according to the ecotypes and soil.

Generally, LP ecotypes exhibited higher contents than LC ecotypes, and sl soil appeared to promote higher isoflavone accumulation than scl. In this regard, LP_af_-sl exhibited the highest significant amounts (*p* < 0.05) compared to the other seed samples (except for the flavone **4**, whose highest content was found to LP_af_-scl (6.7 mg/100 g), whereas the global lowest amount was evidenced to LC_af_-scl (Table 4). On the other hand, in the case of citric acid-debittered LC seed sample (i.e., LC_af_-sl + A), the (iso)flavone contents resulted in a different isoflavone content distribution than the water-based debittering. Significantly highest levels (*p* < 0.05) were obtained for four compounds (i.e., **5**, **6**, **7**, and **8**), but other compounds exhibited the lowest levels among test seed samples (i.e., **1**–**4**, **9**–**11**). In addition, the global trend was visualized with the total isoflavone content (i.e., the sum of the individual contents per compound), whose highest total content was afforded for LC_af_-sl + A (198.3 mg/100 g), and LC_af_-scl yielded the lowest amount (123.6 mg/100 g), comprising significant differences between seed samples.

**Figure 5:**
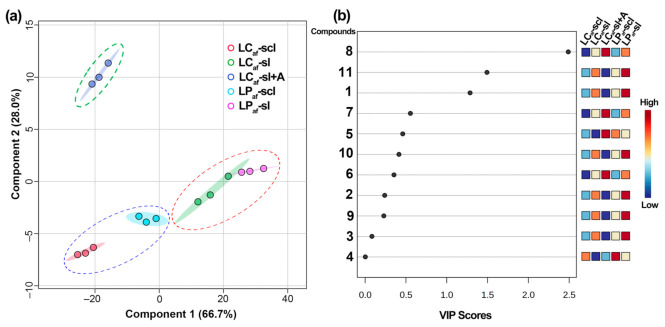
(**a**) PC1 vs. PC2 score plot and (**b**) Variable importance in projection (VIP) plot derived from the partial least squares-discriminant analysis (PLS-DA) on the quantitative dataset of selected (iso)flavonoids from *L. mutabilis* seeds. The PLS-DA model was built using the five groups according to the ecotypes, soil type, and extractant. LC = *L. mutabilis* ecotype from Cajicá; LP = *L. mutabilis* ecotype from Pasto; Subscript af abbreviation = alkaloid-free; LC_af_-sl (LC ecotype grown on silty loam soil); LC_af_-scl (LC ecotype grown on sandy clay loam soil); LP_af_-sl (LP ecotype grown on silty loam soil); LP_af_-scl (LP ecotype grown on sandy clay loam soil); LC_af_-sl + A (LC ecotype grown on silty loam soil and extracted with citric acid). Each colored square on the VIP plot left side indicates the highest (dark red) or lowest (dark green) relation between the seed sample and compound.

The PLS-DA expanded the quantitative data examination of (iso)flavones **1**–**11** to recognize the variability patterns according to ecotypes and soil types (Figure 5). The model performance was checked through a 10-fold cross-validation (CV), implicating an accuracy rate of 60.6% from the two first principal components (PC), which satisfactorily explained the dataset covariance construct. The PC1 × PC2 scores plot exhibited good fitting and predicting parameters (i.e., R^2^_cum_ = 0.947; Q^2^_cum_ = 0.868) and organized the sample set into two main clusters depending on the soil type (Figure 5a), marking the big difference along PC1 (66.7%). In addition, the differences by ecotype and even the debittering approach were mainly explained along PC2 (28.0%).

The respective variable importance in the projection (VIP) plot (Figure 5b) indicated that the discrimination of LP_af_-sl was influenced by the abundance of six isoflavones (**1**–**3**, **9**–**11**), while LP_af_-sl by one compound (**4**), and citric acid-debittered seed sample (i.e., LC_af_-scl + A) by four compounds (**5**–**8**). In contrast, LC_af_-scl and LP_af_-scl have generally related to the lower contents of test (iso)flavones. Compounds **8**, **11**, and **1** exhibited a more significant discriminating influence and differential pattern with VIP values > 1.0.

### 3.5. Total Phenolic Contents of Alkaloid-Free L. mutabilis Seeds

**Figure 6:**
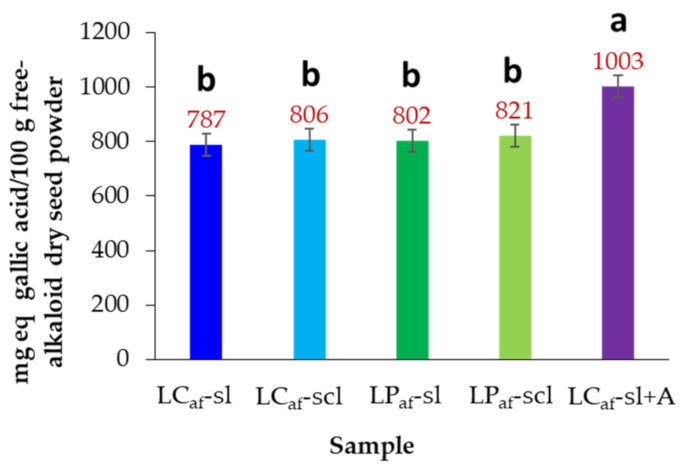
Total phenolic contents of alkaloid-free *Lupinus mutabilis* seed samples. LC = *L. mutabilis* ecotype from Cajicá; LP = *L. mutabilis* ecotype from Pasto; Subscript af abbreviation = alkaloid-free; LC_af_-sl (LC ecotype grown on silty loam soil); LC_af_-scl (LC ecotype grown on sandy clay loam soil); LP_af_-sl (LP ecotype grown on silty loam soil); LP_af_-scl (LP ecotype grown on sandy clay loam soil); LC_af_-sl + A (LC ecotype grown on silty loam soil and extracted with citric acid). Bars represent the mean values ± standard deviation (*n* = 6). Different lowercase letters over bars indicate statistically significant differences according to the post hoc Tukey test (*p* < 0.05).

The total phenolic contents of the test alkaloid-free seed extracts of *L. mutabilis* revealed no significant differences between ecotypes and soils, ranging from 787 to 821 mg/100 g dsp for those extracts obtained from neutral extraction (Figure 6). In contrast, the LC ecotype seeds grown on silty loam soil and debittered with 0.5 M citric acid solution (LC_af_-sl + A) showed a significantly higher phenolic content (21.5% upper) than LC ecotype seeds grown on silty loam soil and debittered conventionally with water (LC_af_-sl) (Figure 6).

### 3.6. Antioxidant Capacity of Alkaloid-Free L. mutabilis Seeds

**Figure 7:**
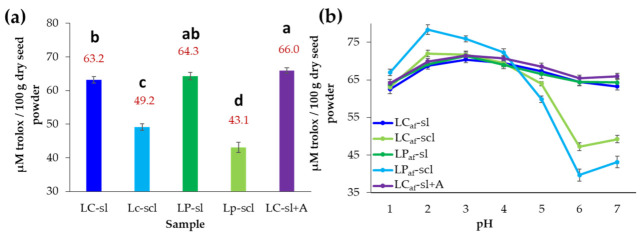
Antioxidant capacity of alkaloid-free *L. mutabilis* seed extracts based on the 2,2-diphenyl-1-picrylhydrazyl (DPPH) radical-scavenging expressed as Trolox equivalent antioxidant activity (TEAC) in µM Trolox per 100 g dry seed powder (µM/100 g dsp). (**a**) TEAC values at neutral pH; (**b**) TEAC values along different acid pH values (1–7). LC = *L. mutabilis* ecotype from Cajicá; LP = *L. mutabilis* ecotype from Pasto; Subscript af abbreviation = alkaloid-free; LC_af_-sl (LC ecotype grown on silty loam soil); LC_af_-scl (LC ecotype grown on sandy clay loam soil); LP_af_-sl (LP ecotype grown on silty loam soil); LP_af_-scl (LP ecotype grown on sandy clay loam soil); LC_af_-sl + A (LC ecotype grown on silty loam soil and extracted with citric acid). TEAC are expressed as mean values ± standard deviation (*n* = 6). Different lowercase letters over bars indicate statistically significant differences according to the post hoc Tukey test (*p* < 0.05).

The antioxidant capacities of the investigated *L. mutabilis* alkaloid-free seed extracts were determined through the DPPH radical-scavenging ability and expressed as TEAC values (µM/100 g dsp). The water-debittered seeds exhibited TEAC values in the range of 43.1–66.0 µM/100 g dsp (Figure 7a), whose highest antioxidant capacity was found for both ecotypes grown in sl soil (63.2 and 64.3 µM/100 g dsp, respectively). No significant differences were found between ecotypes in sl soil. However, a soil effect was observed since those seeds obtained from scl-grown plants exhibited a reduced radical scavenging capacity (<49.2 µM/100 g dsp). The citric acid-debittered seeds showed a significantly higher antioxidant capacity than the LC_af_-sl seeds (66.0 µM/100 g dsp).

The antioxidant capacity of the alkaloid-free seeds exhibited a pH-dependent variation (Figure 7b). However, extracts from seeds harvested from plants grown in sl soil, regardless of ecotype, exhibited a lesser capacity variation along pH values than the extracts from seeds obtained from scl-propagated plants. The highest activity of seeds from sl soil (i.e., LP_af_-sl, LC_af_-sl, and LC_af_-sl + A) resulted in pH 3 (70.4–71.4 µM/100 g dsp), while their lowest radical-scavenging was found at pH 6 (64.4–65.5 µM/100 g dsp). In this case, no substantial variation was observed for the extract derived from citric acid-debittered seeds. Contrarily, seeds from scl soil (i.e., LP_af_-scl and LC_af_-scl) exhibited the highest TEAC values at pH 2 (72.0–78.3 µM/100 g dsp), but a drastic depletion was observed at pH 6 (39.7–47.2 µM/100 g dsp) (Figure 7b).

### 3.7. Iron Content of Alkaloid-Free L. mutabilis Seeds

**Figure 8:**
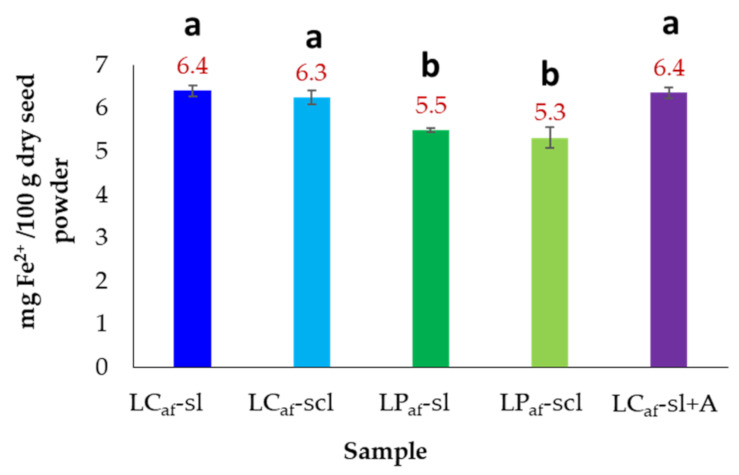
Iron (Fe^2+^) contents of alkaloid-free seeds of *Lupinus mutabilis.* LC = *L. mutabilis* ecotype from Cajicá; LP = *L. mutabilis* ecotype from Pasto; Subscript af abbreviation = alkaloid-free; LC_af_-sl (LC ecotype grown on silty loam soil); LC_af_-scl (LC ecotype grown on sandy clay loam soil); LP_af_-sl (LP ecotype grown on silty loam soil); LP_af_-scl (LP ecotype grown on sandy clay loam soil); LC_af_-sl + A (LC ecotype grown on silty loam soil and extracted with citric acid). Bars represent the mean values ± standard deviation (*n* = 6). Different lowercase letters over bars indicate statistically significant differences according to the post hoc Tukey test (*p* < 0.05).

The iron(II) concentrations for the test *L. mutabilis* seeds were measured and expressed as mg Fe^2+^/100 g dry seed powder (Figure 8). No significant differences were found between seeds from soil types and even those debittered with citric acid (6.4 mg/100 g dsp). However, the iron(II) contents in the LC ecotype seeds (6.3–6.4 mg/100 g dsp) were significantly higher than those from LP ecotype (5.3–5.5 mg/100 g dsp).

### 3.8. Emulsifying Capacity of Lupin Seed-Based Protein

**Figure 9:**
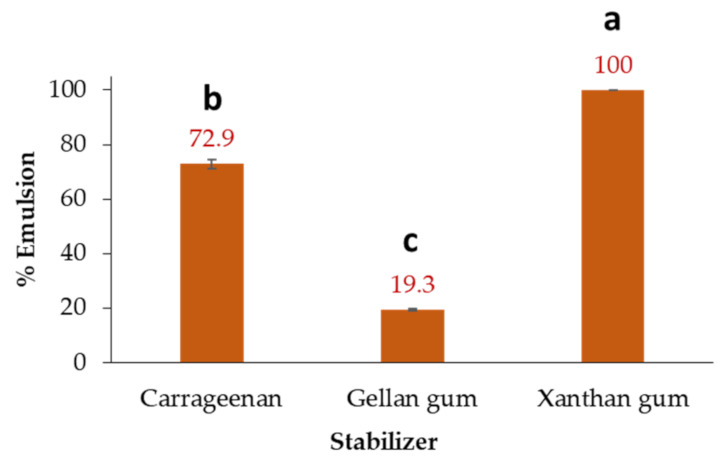
Emulsifying capacity of alkaloid-free *Lupinus mutabilis* LC_af_-sl (*L. mutabilis* from Cajicá ecotype grown on silty loam substrate) seed dispersions after 18 h of preparation. Bars represent the mean values ± standard deviation (*n* = 3). Different lowercase letters over bars indicate statistically significant differences according to the post hoc Tukey test (*p* < 0.05).

The stability of emulsions prepared between coconut oil and water-debittered *L. mutabilis* seeds from LC ecotype plants propagated in sl soils was finally explored. Three biopolymer stabilizers (i.e., carrageenan, gellan gum, and xanthan gum) were employed to assess the emulsifying capacity of the debittered *L. mutabilis* protein. The freshly prepared *L. mutabilis* seed dispersions formed the typical, whitish-colored milk, and the emulsifying capacity was measured after 18 h. Thus, the outcome indicated that the formulation with carrageenan emulsified 72.8% of the coconut fat, whereas gellan gum reached 19.3%, and the seed samples with xanthan gum emulsified 100% fat (Figure 9). Each emulsion was stored at 4 °C and checked 10, 20, 30, and 60 days after its preparation, and no additional floating coconut fat was evidenced, thus confirming the stability of the prepared emulsions.

## 4. Discussion

*L. mutabilis* is an attractive legume plant due to its nutritional and medicinal properties [46]. However, it remains an insufficiently characterized, underexplored, and underemployed plant [26]. In this context, the present study was focused on exploring the variations of nutritional and functional features (i.e., soluble protein and iron contents and antioxidant capacity, phenolic content, and isoflavone profiles) for harvested seeds from two ecotypes of the Andean lupin (*L. mutabilis*) propagated in two soil types commonly found in the Andean highlands, Colombia. LC ecotype showed faster germination during cultivation, involving better growth and an overall condition than LP ecotype. This behavior could happen due to a slow LP ecotype adaptation to the Cajicá cultivation conditions, the seed storing time and/or conditions, and genetic factors [47,48].

The first evidenced variations after propagation were related to the color and length of pods and dry weight and diameter of seeds since they differed depending on the ecotype and the soil type. Such phenotypic variations have been previously documented for indeterminate-growth genotypes of *L. mutabilis* cultured in the Andean highlands and even Europe owing to its high genetic variability and plasticity [49], having broad adaptive properties to diverse environments depending on water availability, climate, and soil types [50,51]. In this regard, LC ecotype initially showed higher seed dry weight and diameter than LP ecotype, and these differences remained until propagation in silty loam (sl) and sandy clay loam (scl) soils, although sl promoted a size increase (>6%) in LP ecotype. In addition, seeds harvested from scl-grown plants tended to have smaller pod size and seed dry weight and diameter than sl-grown seeds due to the lower humidity saturation, organic matter, and total nitrogen between scl and sl soils, and even the waterlogging possibility in sandy clay soils which affects seed production, root structure, and plant growth [26,51]. These phenotypic variations suggested that the chemical characteristics could vary by soil type effect, which was further explored to characterize the seeds from these two ecotypes. In this regard, the effect of soil features on the nutritional properties of the test Andean lupin ecotypes was globally measured using two different Andean soil types. These soil-dependent variations are not well understood for the Andean lupin, and no soil effect has been determined for its isoflavone content and other parameters. However, our findings determined that the investigated *L. mutabilis* ecotypes showed differential isoflavone contents in their collected seeds. Further studies will be conducted to define the specific effect of certain soil features on the measured content to provide a mechanistic perspective of such a soil effect.

The optimal nutritional status of any person varies depending on age, gender, physical activity, size, and even the climate where they live. The diet must cover basic needs for dietary energy, protein, minerals, and vitamins [52]. Currently, the recommended daily protein intake for adults over 19 years of age (except for pregnancy and lactation) is 0.8 g per kg per day [53]. For this reason, high-protein food is highly valued. Lupin seed proteins are categorized as globulins and albumins, in a 9:1 ratio, which are water and salt soluble, respectively [54]. Therefore, soluble proteins constitute the main protein fraction in lupin seeds. In this regard, the soluble protein analysis by the Bradford method of test seeds from the two *L. mutabilis* ecotypes revealed a high soluble protein content (45–49% range, Table 2). These values are very similar to those reported contents (41–49% range) for *L. mutabilis,* determined by the Kjeldahl method (protein = nitrogen content × 6.25), grown in southern Colombia [7] and varieties analyzed in Poland [55]. The differences in protein contents between ecotypes and varieties can be attributed to the fact that, as above-mentioned, *L. mutabilis* has an important genetic variability leading to adapting to soil and climate characteristics, which also causes differences in the seed protein and oil contents [26,50].

There was a reduced seed protein accumulation trend for LP ecotype and scl soil, but no significant differences were found according to the tested factors. Therefore, these investigated ecotypes suit lupin protein sources cultivated in both soils. However, the results of *L. mutabilis* raw seed characterization revealed that LC and LP ecotypes have an appreciable alkaloid content that must be removed before further analysis since these compounds have anti-nutritional properties [56]. Alkaloids have been previously reported in *L. mutabilis* seeds from plants collected in Ecuador [30] or Peru [57,58]. A scale was created in Poland for lupin plants for comparative purposes according to the total alkaloid content (TAC), comprising very bitter (TAC > 1%), intermediate bitter (0.3% < TAC < 1%), low bitter (0.15% < TAC < 0.3%), and very low bitter (TAC < 0.15%) [59]. According to the TAC values of the test *L. mutabilis* ecotypes (TAC < 0.23%), they can be considered low bitter seeds. Therefore, alkaloids were removed to afford a negligible TAC below LoD (i.e., 0.06 mg/100 g dsp), requiring an improved debittering program (>139 h and <7.0 L water) to comply with the international regulations for safety limit to human consumption (≤20 mg/100 g dsp) [60]. Thus, a subsequent characterization of these debittered seeds might be conducted to examine their properties as food raw material. In addition, although our debittering program required more time (>139 h) to deplete the alkaloid content below Dragendorff’s test LoD in comparison to previous studies [57], the water volume consumption was reduced (4.9 to 7.0 L for 100 g of seeds) due to the lower alkaloid content in LP and LC ecotypes (i.e., TAC < 0.23%).

The subsequent analysis on alkaloid-free seeds showed that the average protein content of test seeds showed a depletion after water-based debittering (ca. 10% loss, Table 2). However, despite this reduction, these contents are comparable with raw materials widely used in the food industry, such as soybeans, whose improved varieties reached a 40% dry matter basis [61]. In contrast, citric acid-mediated debittering afforded seed material with lesser protein content (i.e., 24.3%). A recent previous study describing a structured water change program for alkaloid removal (80% reduction) on intermediate-bitter seeds (i.e., TAC > 0.35%) of three *L. mutabilis* varieties (employing water or 0.5% saline water, 11 to 7-time intervals, and 87–58 h) involved ca. 6% total nitrogen loss [57]. Although the soluble protein content and total nitrogen are different variables related to seed soluble protein, such a protein loss in *L. mutabilis* during debittering can be rationalized by protein solubility increase as a consequence of a diminished number of protein–protein interactions and thermal stability, particularly in the hydration and cooking steps [57,62]. The protein losses were accompanied by dry matter loss (27–38%), a common outcome of seed debittering. In this context, concerning alkaloid elimination, the most efficient debittering process was performed with citric acid since the processing time was shorter, as reported for *L. albus* [63]. However, this debittering has drawbacks because of the higher protein and dry matter losses. Therefore, considering the nutritional importance of protein, the traditional water-based alkaloid removal method was considered a better choice because it generated lower protein losses than the citric acid-mediated treatment.

Additionally, due to the high soluble protein content in debittered seeds (>36%), the emulsifying capacity was then measured to define the coconut oil amount that protein can emulsify before phase inversion. Thus, refrigerated storage revealed that the powdered seed formulation of *L. mutabilis* protein and xanthan gum achieved stable emulsions with 1.5% coconut oil. Combining Andean lupin protein with the other two stabilizers showed a lower emulsifying capacity, so the carrageenan mixture was 27.1% below, and gellan gum only reached 19.3% of the xanthan gum emulsifying capacity. The rheological effect of xanthan polymer on the emulsifying capacity of Andean lupin protein is due to the xanthan pseudoplastic nature, adding elasticity and even antioxidant properties and viscous performance for improving the texture, appearance, and quality of the drink formulation [64]. Therefore, those emulsions prepared with *L. mutabilis* seed protein and xanthan gum are like those widely used in the food industry [38,39].

Although the seed samples exhibited very similar LC-MS-derived isoflavone profiles, the total contents ranged between 123 to 198 mg/100 g dsp. This fact indicated that the ecotype variation and soil effect on isoflavone content were manifested in abundance instead of occurrence. The citric acid-based treatment also produced the lowest isoflavone content for seven compounds. However, the highest levels for the other four compounds after citric acid-based debittering were possibly due to the matrix effect and dry matter loss. This fact suggested that citric acid treatment led to a faster alkaloid removal but also can affect the isoflavone level distribution to be extracted in debittered seeds, resulting in a higher total content (198 mg/100 g). The measured contents for *L. mutabilis* seeds were higher than those reported for Andean lupin varieties from Brazil and Peru (<35 mg/100 g) [6]. However, isoflavones quantification carried out in Brazil on 14 soybean varieties revealed that the variety with the highest content was 188 mg/100 g fresh seed (fs), and the lowest was 57 mg/100 g fs [65]. Although the contents measured in the present study were obtained after debittering and dry basis, our findings indicated that the test seeds from these *L. mutabilis* ecotypes are a good isoflavone source.

The scl soil seemed to affect the isoflavone accumulation in seeds since the measured contents were generally lower than those from sl soil. The lower humidity saturation, organic matter, and nitrogen availability of scl soil can be the critical factors for decreasing the isoflavone content since these factors can affect the isoflavone production and accumulation, especially temperature, moisture, and nutrition [66,67]. Considering that some isoflavones play an essential role in building a robust mutualistic relationship with rhizobia [68] and environmental and geographical conditions can promote changes in the isoflavone accumulation [67], the information on the effects of chemical and microbiological soil composition should be expanded to disclose the mechanisms for isoflavone accumulation in *L. mutabilis* seeds.

The antioxidant capacity of the test seed samples followed a similar pattern since a soil-dependent variation was observed. This fact can be explained since isoflavones are well-known to exhibit strong antioxidant properties [69], so this trend agrees with the high-common relationship between isoflavone content and antioxidant properties of soybean seeds [70,71]. Consequently, depending on the soil type, both ecotypes appeared to have the same storage tendency for antioxidant isoflavones. In contrast, although phenolic compounds exhibit antioxidative actions [72], the total phenolic content did not change depending on the ecotype and soil type. However, citric acid-treated seeds showed the highest total phenolic content and radical scavenging, suggesting a major matrix retention of antioxidant compounds under acid-based debittering conditions.

Additionally, the antioxidant capacity was affected by the assay pH 1 to 7. Some phenolic compounds (e.g., phenolic acids, (iso)flavonoids, phenylpropanoids) can change the phenolic structure irreversibly and significantly modify antioxidant properties [73,74]. Thus, the antioxidant capacity of sl-propagated seed samples was not substantially modified by the pH (i.e., 62 < TEAC < 71 µM/100 g) but notably higher to those seed extracts from scl-grown plants, revealing a relevant performance, mainly for LP ecotype propagated in scl soil (40 < TEAC < 78 µM/100 g). This trend can be attributed to those compounds having a pH-dependent structural alteration to modulate the antioxidant capacity, especially at very acidic pH. However, the DPPH assay is very sensitive to acidic pH values, so the radical-scavenging ability can be considered highly variable and unreliable below pH 4 [75] because an increased acidity reduces the DPPH/antioxidant reaction rate [76]. This pH-dependent antioxidant performance harmonizes our results for improved food processing decisions, especially during commercial production.

The iron content was finally determined to explore another nutritional feature of the *L. mutabilis* seed as raw food material. The human body uses iron for various physiological processes, and it must be ingested from edible sources to maintain an adequate nutritional status [21]. The iron content in LP seed was 13.5% below the informed content of an *L. mutabilis* ecotype in southern Colombia, while the iron levels in LC seed were 0.9% above [7]. In addition, LC ecotype was found to contain 60% more iron than lupin varieties grown in Peru [77]. Compared to foods for daily consumption, LC and LP ecotypes contain 81.4% and 54.5% more iron than beef, 154% and 116.4% more iron than eggs, and 5% and 170.5% more iron than fish and chicken, respectively [78,79]. Finally, our findings revealed that iron content between water- and citric acid-debittered LC seeds was not significantly different after alkaloid removal, suggesting that iron levels can be retained after exposure to an acidic medium. This observation agreed with an analysis in Ecuador, which showed that the amount of iron is stable during the processing of *L. mutabilis* seed [30].

## 5. Concluding Remarks

The characterization of debittered seeds from two Andean lupin (*L. mutabilis*) ecotypes was successfully achieved regarding nutritional (soluble protein and iron) and functional (antioxidant capacity, phenolic content, and isoflavone profiles) properties. In this regard, being native to the Andes, *L. mutabilis* found in Colombia was propagated in the Bogotá plateau, in two different soil types and without fertilizer, to produce seeds with a high soluble protein content (>40%). However, the tested *L. mutabilis* ecotypes (LP and LC) produced low-bitter seeds (i.e., 0.15% < TAC < 0.3%), which required an alkaloid removal process before further employing as food raw material suitable for human or animal consumption. The citric acid can shorten the processing time and water volume, resulting in higher dry matter and protein losses, so water-based debittering was the preferred method. Additionally, propagated plants produced seeds that, after conventional debittering, exhibited attractive contents of soluble protein (24–39 g/100 g dry seed powder (dsp)), phenolic (787–1003 g/100 g dsp), isoflavone (1–104 g/100 g dsp), and iron (5.3–6.4 g/100 g dsp), as well as TEAC-based antioxidant capacity (39–78 µM/100 g dsp). Furthermore, the *L. mutabilis* seed protein was able to emulsify 1.5% coconut oil stabilized by xanthan gum, which is highly favorable due to the viscosity and antioxidant properties of this stabilizer.

Slight differences were found in the soluble protein content of raw seeds harvested from plants grown on silty loam or sandy clay loam soils, but no significant differences were found in the protein content of debittered seeds. In addition, higher pH, humidity saturation, organic matter, and total nitrogen of silty loam soil seemed to promote isoflavone accumulation and better antioxidant capacity at pH 4–7, and, in contrast, no soil effect was observed for total phenolic and iron contents. Additionally, LC ecotype exhibited higher seed diameter, seed biomass, and iron content, while LP ecotype showed higher isoflavone levels. This study is the first report on the nutritional and functional characterization of debittered seeds from two ecotypes of Colombian Andean lupin involving global soil effects. The entire set of results is very attractive, considering that the present study seeks to determine the potential of the test *L. mutabilis* ecotypes as food raw material. Thus, based on the measured properties, the findings revealed that the investigated debittered seeds might be used to develop an Andean lupin-based edible product. However, further chemical, molecular, and agronomical characterization is needed to complete the relevant traits for its agricultural opportunities and food applications.

## Figures and Tables

**Table 1:** Pod and seed variations of propagated *L. mutabilis* ecotypes in two soil types.

	Silty Loam (sl) Soil		Sandy Clay Loam (scl) Soil	
	LC ^a^	LP ^b^	LC ^a^	LP ^b^
Pod size (cm)	8.1 ± 1.8 ^A^	6.5 ± 1.3 ^A^	7.3 ± 1.6 ^A^	5.7 ± 1.4 ^A^
Seed dry weight (g)	0.39 ± 0.04 ^A^	0.31 ± 0.03 ^B^	0.33 ± 0.02 ^AB^	0.25 ± 0.02 ^C^
Seed diameter (mm)	9.9 ± 0.3 ^A^	9.2 ± 0.6 ^AB^	9.7 ± 0.4 ^AB^	8.9 ± 0.4 ^B^

^a^ LC = *L. mutabilis* ecotype from Cajicá; ^b^ LP = *L. mutabilis* ecotype from Pasto. Data expressed as mean values ± standard deviation (*n* = 30). Different superscript uppercase letters indicate statistically significant differences according to the post hoc Tukey test (*p* < 0.05).

**Table 2:** Seed soluble protein of *L. mutabilis* raw and alkaloid-free from two ecotypes.

Samples ^a^	Ext ^b^	RCT ^c^	WV ^d^	PC_i_ ^e^	PC_f_ ^f^	PL ^g^	DML ^h^	TAC_i_ ^i^	TAC_f_ ^j^
LC-sl	W	195	7.0	48.9 ± 0.7	39.1 ± 1.7	9.8	27.5 ± 1.3	236.5 ± 2.4	<LoD ^k^
LC-scl	W	187	6.7	47.1 ± 0.7	37.7 ± 1.6	9.4	27.4 ± 0.9	190.5 ± 1.9	<LoD ^k^
LP-sl	W	187	6.7	47.4 ± 0.8	38.8 ± 1.1	8.6	32.1 ± 1.1	187.6 ± 2.5	<LoD ^k^
LP-scl	W	187	6.7	45.3 ± 0.7	36.6 ± 1.5	8.7	31.3 ± 1.3	175.4 ± 2.1	<LoD ^k^
LC-sl + A	A	139	4.9	48.9 ± 0.7	24.3 ± 0.7	24.6	38.1 ± 1.5	206.5 ± 2.4	<LoD ^k^

^a^ LC = *L. mutabilis* ecotype from Cajicá; LP = *L. mutabilis* ecotype from Pasto; LC-sl (LC ecotype grown on silty loam soil); LC-scl (LC ecotype grown on sandy clay loam soil); LP-sl (LP ecotype grown on silty loam soil); LP-scl (LP ecotype grown on sandy clay loam soil); LC-sl + A (LC ecotype grown on silty loam soil and extracted with citric acid). Different lowercase letters over bars indicate statistically significant differences according to the post hoc Tukey test (*p* < 0.05); ^b^ Ext = extractant: W = distilled water; A = 0.5 M citric acid solution; ^c^ RCT = time (in h) elapsed for alkaloid removal; ^d^ WV = total water volume (in liters (L)) employed for alkaloid removal of 100 g of seeds; ^e^ PC_i_ = initial protein content (%) before debittering (Figure 2a); ^f^ PC_f_ = final protein content (%) after debittering; ^g^ PL = protein loss (%) after debittering (Figure 2b); ^h^ DML = dry matter loss (%); ^i^ TAC_i_ = initial total alkaloid content (mg eq lupanine/100 g dry seed powder (dsp)) before debittering; ^j^ TAC_f_ = final total alkaloid content after debittering below the limit of detection (LoD) of Dragendorff’s test; ^k^ LoD of Dragendorff’s test = 0.06 mg eq lupanine/100 g dsp. Data expressed as mean values ± standard deviation (*n* = 6).

**Table 3:** Annotated compounds by LC-MS in alkaloid-free seed extracts of *L. mutabilis* ecotypes.

# ^a^	t_R_ (min) ^b^	[M + H]^+^ *m*/*z*	[M − H]^−^ *m*/*z*	Accurate Mass	Error ^c^	Formula	λ_max_^d^	Annotation ^e^
**1**	2.8	305	303	303.0512	2.3	C_15_H_10_O_7_	275,329	dihydroxygenistein
**2**	2.9	319	317	317.0674	4.1	C_16_H_12_O_7_	271,331	dihydroxymutabilein
**3**	4.5	289	287	287.0569	4.5	C_15_H_10_O_6_	269,329	hydroxygenistein
**4**	8.8	273	271	271.0614	3.0	C_15_H_10_O_5_	267,339	apigenin
**5**	14.4	273	271	271.0611	1.8	C_15_H_10_O_5_	262,328	genistein
**6**	16.3	303	301	301.0721	3.0	C_16_H_12_O_6_	270,326	hydroxymutabilein
**7**	16.5	317	315	315.0855	−4.4	C_17_H_14_O_6_	264,323	methoxymutabilein
**8**	17.6	287	285	285.0751	−4.2	C_16_H_12_O_5_	266,325	mutabilein
**9**	21.8	373	371	371.1146	4.0	C_20_H_18_O_7_	277,329	lupinisoflavone D or B
**10**	22.0	357	355	355.1173	−2.5	C_20_H_18_O_6_	278,331	luteone
**11**	32.6	341	339	339.1246	4.1	C_20_H_18_O_5_	278,330	lupiwighteone

^a^ Compound numbering according to Figure 4; ^b^ t_R_ = retention time (min); ^c^ Relative error (in ppm) between HRMS-measured accurate mass and theoretical monoisotopic mass of the quasimolecular ion; ^d^ Ultraviolet maximum absorption (λ_max_) evidenced for the band II and I of (iso)flavonoids by liquid chromatography coupled with a photodiode detector analysis; ^e^ Annotated (iso)flavones at level 3 according to the confidence levels to communicate metabolite identity by high-resolution mass spectrometry (HRMS) [42].

**Table 4:** Variation in the profile of isoflavones in alkaloid-free *L. mutabilis* seeds.

# ^a^	LC_af_-sl ^b^	LC_af_-scl ^b^	LP_af_-sl ^b^	LP_af_-scl ^b^	LC_af_-sl + A ^b^	
**1**	23.2 ± 1.0 ^B^	18.5 ± 0.6 ^D^	25.5 ± 1.1 ^A^	21.5 ± 1.1 ^C^	17.4 ± 0.5 ^D^	
**2**	12.2 ± 0.6 ^A^	9.6 ± 0.3 ^C^	12.4 ± 0.4 ^A^	10.7 ± 0.3 ^B^	9.2 ± 0.2 ^C^	**Hm** ^c^
**3**	4.2 ± 0.1 ^A^	3.5 ± 0.1^C^	4.4 ± 0.2 ^A^	3.8 ± 0.1 ^B^	1.9 ± 0.1 ^D^	H
**4**	4.8 ± 0.1 ^C^	6.0 ± 0.2 ^B^	5.0 ± 0.2 ^C^	6.7 ± 0.2 ^A^	4.9 ± 0.2 ^C^	
**5**	15.5 ± 0.7 ^C^	16.3 ± 0.7 ^C^	18.4 ± 0.6 ^B^	18.9 ± 0.6 ^B^	22.4 ± 0.5 ^A^	
**6**	3.2 ± 0.1 ^C^	1.4 ± 0.1 ^D^	4.4 ± 0.1 ^B^	3.0 ± 0.1 ^C^	6.2 ± 0.3 ^A^	M
**7**	5.1 ± 0.1 ^C^	2.7 ± 0.1 ^E^	7.8 ± 0.4 ^B^	4.0 ± 0.1 ^D^	17.1 ± 0.6 ^A^	
**8**	51.0 ± 3.0 ^C^	38.3 ± 1.6 ^E^	61.0 ± 1.6 ^B^	47.5 ± 1.3 ^D^	104.3 ± 4.6 ^A^	
**9**	2.4 ± 0.1 ^B^	1.3 ± 0.1 ^C^	3.2 ± 0.1 ^A^	2.4 ± 0.1 ^B^	0.8 ± 0.1 ^D^	L
**10**	5.6 ± 0.2 ^B^	3.5 ± 0.1 ^C^	7.0 ± 0.1 ^A^	5.5 ± 0.1 ^B^	3.1 ± 0.1 ^D^	
**11**	26.7 ± 0.5 ^B^	22.6 ± 1.1 ^C^	32.1 ± 1.2 ^A^	26.5 ± 1.0 ^B^	11.0 ± 0.3 ^D^	
Total ^d^	154.0 ± 6.6 ^C^	123.6 ± 4.8 ^D^	181.4 ± 5.6 ^B^	150.6 ± 4.9 ^C^	198.3 ± 7.3 ^A^	

^a^ Compound numbering according to Figure 4 and compound annotations according to Table 3; ^b^ (iso)flavone concentrations expressed as mg genistein equivalents per 100 g of alkaloid-free dry seed powder (mg/100 g dsp) per *L. mutabilis* ecotype. LC = *L. mutabilis* ecotype from Cajicá; LP = *L. mutabilis* ecotype from Pasto; Subscript af (_af_) abbreviation = alkaloid-free; LC_af_-sl (LC ecotype grown on silty loam soil); LC_af_-scl (LC ecotype grown on sandy clay loam soil); LP_af_-sl (LP ecotype grown on silty loam soil); LP_af_-scl (LP ecotype grown on sandy clay loam soil); LC_af_-sl + A (LC ecotype grown on silty loam soil and extracted with citric acid); ^c^ Hm = heatmap color scale: H =highest value, M = medium value, L = lowest value; ^d^ Total isoflavone content as the sum of the individual isoflavone contents per seed sample. Data expressed as mean values ± standard deviation (*n* = 6). Different uppercase letters along rows (i.e., compounds) indicate statistically significant differences according to the post hoc Tukey test (*p* < 0.05).

## Data Availability

The data presented in this study are available from the authors upon reasonable request.

## Appendix A

**Table A1:** Characteristics of the test soils employed to propagate *L. mutabilis* ecotypes.

Characteristic	Sandy Clay Loam (scl) Soil	Silty Loam (sl) Soil
pH	4.79	5.76
Electric conductivity (dS/m)	0.22	0.29
Cation exchange capacity (meq/100 g)	11.9	14.4
Mean humidity saturation (%)	23.9	41.9
Oxidable organic carbon (%)	0.708	9.72
Organic matter (%)	1.22	16.8
Total nitrogen (%)	0.059	0.810
Apparent density (g/cm^3^)	0.956	0.616
Clay texture (%)	32.0	18.0
Sand texture (%)	50.0	14.0
Silt texture (%)	18.0	68.0
